# Survey based assessment of diagnosis through periapical radiograph and CBCT and treatment of root resorption with Brazilian and American dentists and endodontists

**DOI:** 10.4317/jced.57774

**Published:** 2021-08-01

**Authors:** Christine-Men Martins, Adrielly-Regina de Moraes, Ana-Julia-Menoti Cruz, Lalleska-Caroline-Pereira Barboza, Victor-Eduardo-de Souza Batista, Graziela-Garrido Mori, Rosana-Leal do Prado, Janine Matos, Bruno Herrera, Priscila-Bruna-Gonçalves Lacerda, Ana-Cristina Andrada

**Affiliations:** 1 DDS, MSc, PhD. Professor at Dental School of Presidente Prudente, University of Western São Paulo - UNOESTE, Presidente Prudente, Sao Paulo, Brazil; 2 Undergraduate at Dental School of Presidente Prudente, University of Western São Paulo - UNOESTE, Presidente Prudente, Sao Paulo, Brazil; 3 Graduate at University of Detroit Mercy School of Dentistry, Detroit, Michigan, USA; 4 Professor at University of Detroit Mercy School of Dentistry, Detroit, Michigan, USA; 5 MsC candidate at Dental School of Presidente Prudente, University of Western São Paulo - UNOESTE, Presidente Prudente, Sao Paulo, Brazil

## Abstract

**Background:**

This study assesses and compares the knowledge level of endodontists (ENDs) and general dental practitioners (GPs) from Brazil and United States of America (USA) in the diagnosis and treatment of internal and external inflammatory root resorptions through periapical radiographic (PA) and cone beam computed tomography (CBCT) examinations.

**Material and Methods:**

A cross-sectional online questionnaire-based survey was presented to the volunteers containing questions regarding personal and professional profile, as well as three clinical cases of internal and external inflammatory root resorption. A series of multiple-choice questions about the diagnosis and treatment options were surveyed. The data collected was analysed by the Chi-square test with Yates correction with a significance level of 5 %.

**Results:**

Most answers were considered adequate when all three questions about the diagnosis and all two questions relating to the treatment were answered accurately. A total of 374 dentists answered the survey (n: 229 from Brazil vs. 145 from USA) being 41% END and 59% GP. END presented higher level of knowledge than GP regarding to diagnosis and treatment of inflammatory root resorptions both in Brazil and USA (*p*<0.05); USA presented higher level of adequate responses than Brazil (*p*<0.05).

**Conclusions:**

END achieved a level of knowledge of the diagnosis and treatment of root resorption superior to the GP. Comparing the results obtained in both countries, it was observed that the USA had a higher correct response rate than Brazil.

** Key words:**Internal root resorption, external root resorption, management, diagnosis, treatment.

## Introduction

Root resorption occurs especially as a result of osteoclastic cell activity (1). In permanent dentition, this is a pathologic event that might lead to tooth loss (2). It can be found on the wall of the root canal (internal resorption) and on the external surface of the root (external resorption or cervical resorption) and it may be transient or progressive (3).

Internal root resorption is an inflammatory process initiated within the pulp space that might lead to a possible cementum invasion. It is followed by a multinucleated giant cells accumulation and, consequently, a granular tissue formation (4). Clinically, it is difficult to diagnose due to its asymptomatic characteristic. However, with the disease progression, chromatic alterations can be noted in the crown of the affected tooth (4). Its diagnosis is usually made through routine radiographic examination, when an uniform oval shaped radiolucent lesion with well-defined and symmetrical contours are observed (3-5). The main etiological factor is dental trauma (5), however dental caries, invasive restorative procedures, and idiopathic factors can also be a factor (2-5). Its progression persists as long as the stimulus is present (2-5). Therefore, after being properly diagnosed, tooth with internal resorption should undergo endodontic treatment in order to remove granulation tissue and blood supply from the resorptive cells (2,6). Sodium hypochlorite irrigation with ultrasonic device should be used because of its characteristic to dissolve the remaining vital tissue (4,7). In addition, intracanal calcium hydroxide-based medication could be used to help control the bleeding and necrotize residual pulp tissue (4,5). If a communication between the root canal and external periodontal tissue is present, the use of bioceramics or MTA is indicated (4).

On the other hand, external inflammatory root resorption occurs as an imbalance between osteoblasts and osteoclasts present in the periodontal ligament (9). Since its progression occurs from the external surface of the roots, in some cases, pulp tissue is found intact (9,10). Its etiology includes: orthodontic movements, chronic periapical lesions, occlusal trauma, dental trauma, orthognathic surgery, periodontal treatment and teeth whitening (11). It is asymptomatic and advanced lesions can have signs of mobility, fracture, and pink discoloration of the crown (12). Radiographically, it presents with irregular limits, variations in density within the lesion, and, most of the times, visible root canal walls (10). If the root canal is affected or the tooth presents with a chronic periapical lesion due to necrosis of the pulp, root canal therapy is indicated (9,12). When the etiology is removed, the affected surface returns to its physiological form due to the cementoblast activity on the external surface of the tooth (9).

As a result of the similarities between internal and external inflammatory root resorptions and their detection being based on radiographic findings, the differential diagnosis should be achieved with multiple radiographs with different angulation for comparison and analysis of the lesions. In addition, dentists are encouraged to use CBCT exams to diagnose and assess the lesions extent and involvement (13).

Proper diagnosis is paramount for their proper treatment. Therefore, the purpose of this study was to assess and compare the knowledge level of endodontists (ENDs) and general dental practitioners (GPs) from Brazil and United States of America (USA) in the diagnosis and treatment of internal and external inflammatory root resorptions through periapical radiographic (PA) and cone beam computed tomography (CBCT) examinations.

## Material and Methods

-Research design 

The project was approved by the Research Ethics Committee of the University of Oeste Paulista - UNOESTE, CAAE 87440618.1.0000.5515 and by the University of Detroit Mercy Institutional Review Board under IRB protocol number 1819-36.

In order to access the study population knowledge, an online questionnaire was designed with a mixture of qualitative and quantitative questions. Dentists were approached through an email database, social networking groups and personal contacts to participate in the research through an open google document. The online survey was kept available for 60 days.

The inclusion criteria were being a private practice dentist in Brazil or USA. If the dentist was a specialist, only endodontist graduated from Brazil or USA was accepted. Subjects not included in these criteria, was excluded from the study.

**Figure 1:**
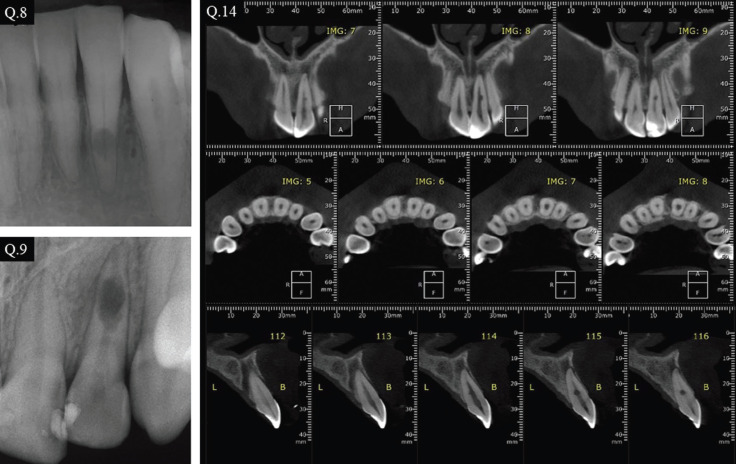
Images of the cases presented in the questionnaire. Q.8: radiograph representing external root resorption; Q.9: radiograph representing internal root resorption; Q.14: CBCT representing internal root resorption.

The questionnaire included a personal and professional profile, such as age, state of practice, experience as a dentist and/or an endodontist, judgment about the quality of endodontic teaching during graduation and the number of cases including internal and/or external resorption observed throughout their professional experience. Subsequently, the volunteer was presented with PA images of clinical cases of internal and external inflammatory root resorption, as well as a CBCT for the internal root resorption case. A series of multiple-choice questions about the diagnosis and treatment of both cases were asked. The survey is shown in Table 1,  Table 1 cont. and Figure 1.

**Table 1:**
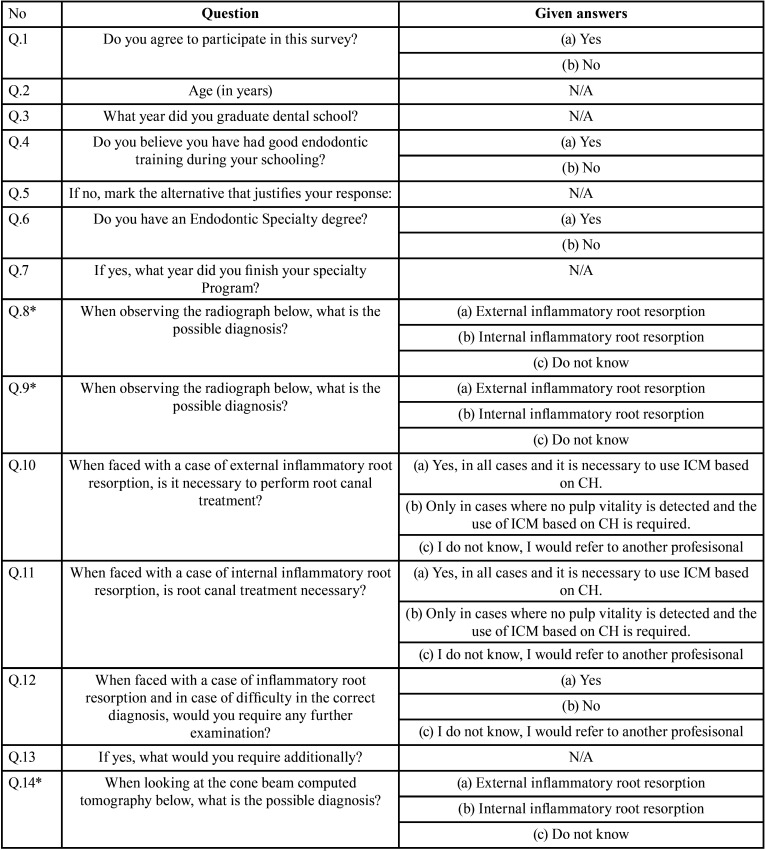
Questionnaire used to evaluate diagnosis and treatment for internal and external root resorption within END and GP.

**Table 1 cont.**
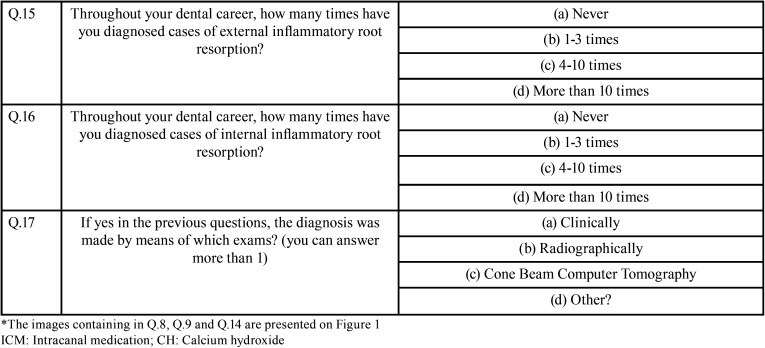
Questionnaire used to evaluate diagnosis and treatment for internal and external root resorption within END and GP.

-Statistical analysis 

The null hypothesis tested is that the knowledge level of general dentists is equal to the endodontists regarding the diagnosis and treatment of inflammatory root resorptions, both in Brazil and USA. The data collected was tabulated and analysed by the Chi-square test with Yates correction by the R software with a significance level of 5 % to compare the differences between specialists or not and within Brazil and USA.

## Results

-Demographics 

Due to the questionnaire distribution, through an email database, social networking groups and personal contacts, we do not know how many subjects had access to the survey. A total of 374 dentists answered the survey (n: 229 from Brazil vs. 145 from USA). Of those, 154 (41%) were ENDs (n: 96, 42% vs. 58, 40% subjects), with a mean age of 42.4±11.8 (41.2 vs. 45) years old, and with an average of 19±12.1 (19.2 vs. 18.8) years of experience. In addition, 220 (59%) were general dentists (n: 133, 58% vs. 87, 60% subjects), with a mean age of 36.3±13.6 (31.3 vs. 44.5) years old, and with an average of 13.1±12.9 (9.7 vs. 18.7) years of experience. All results presented in parenthesis are Brazil vs. USA, respectively (Table 2).

**Table 2:**
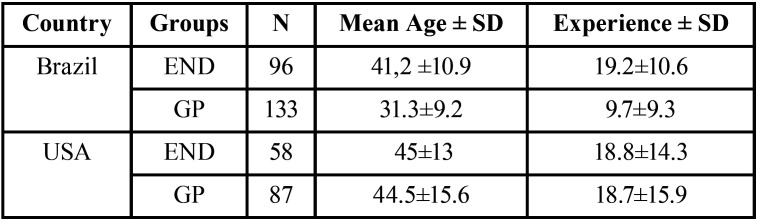
Demographics of the subjects analyzes in this study.

A total of 293 (83.8% vs. 71%; Brazil and USA, respectively) dentists reported having adequate endodontic training during their dental school. When they were asked about their weaknesses, both groups pointed out that lack of self-interest, lack of patients, and outdated instructors were important factors (data not-shown).

Most professionals never diagnosed or diagnosed few cases of internal and external inflammatory root resorption (Table 3), with 82 volunteers diagnosed only with clinical and radiographic examinations and 75 using cone-beam computed tomography.

**Table 3:**
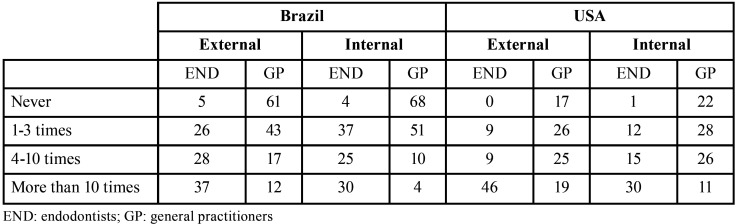
Quantity of diagnosed cases of internal and external root resorption during voluntieers dental carrier.

Diagnosis and treatment results 

**Figure 2:**
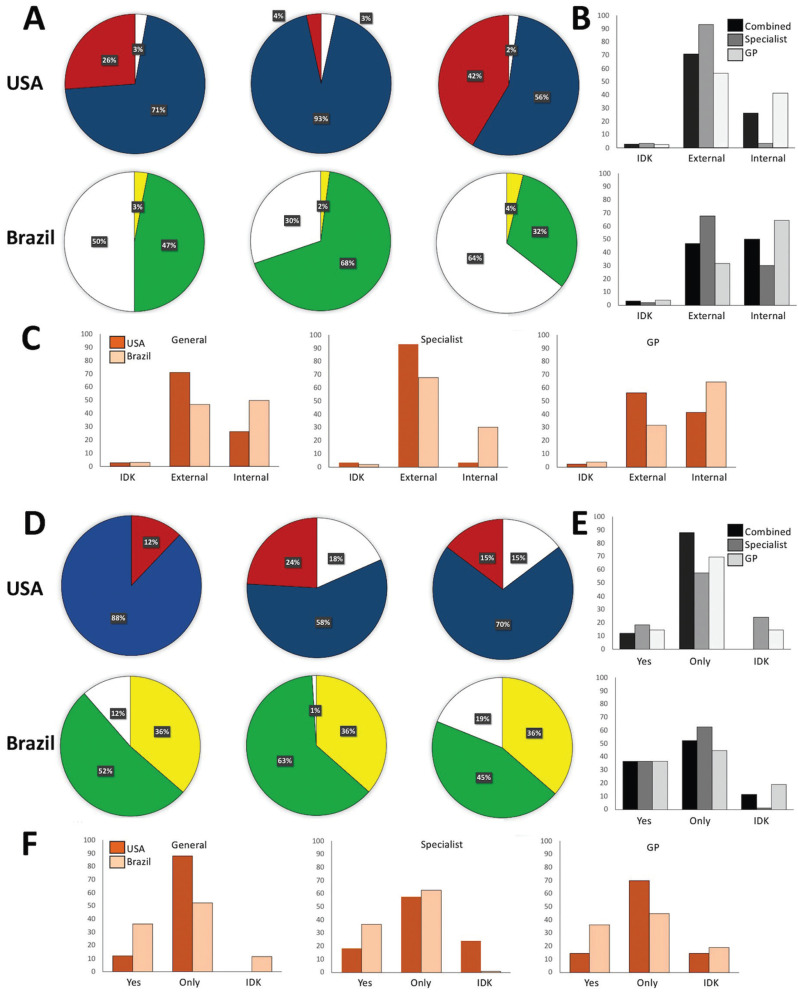
Analysis of the questionnaire about external inflammatory root resorption. Panel A: Comparison of knowledge level between all groups (USA: top pie chart, and Brazil: bottom pie chart) on the diagnosis of external inflammatory root resorption. Blue and green are the correct answers. Panel B: The results organized by individual answer Panel C: The results comparing USA vs. Brazil. IDK (I don’t know), external, and internal root resorption. Panel D: Comparison of knowledge level between all groups (USA: top pie chart, and Brazil: bottom pie chart) on the treatment of external inflammatory root resorption. Top pie chart: USA, and bottom pie chart: Brazil. Blue and green are the correct answers. Panel E: The results organized by individual answer. Panel F: The results comparing USA vs. Brazil. Yes, only, IDK (I don’t know).

Case 1, external root resorption (Fig. 1, Q.8): Overall 56.1% of the surveyed population gave a proper diagnosis, that comprises 67.7% of ENDs and 31.8% of general dentists. In terms of its treatment, 89.7% of ENDs and 57.5% of general dentist would correctly perform endodontic treatment only where no pulp vitality was detected. They agreed that the use of calcium hydroxide intracanal medication would be required on this case (Fig. 2).

**Figure 3:**
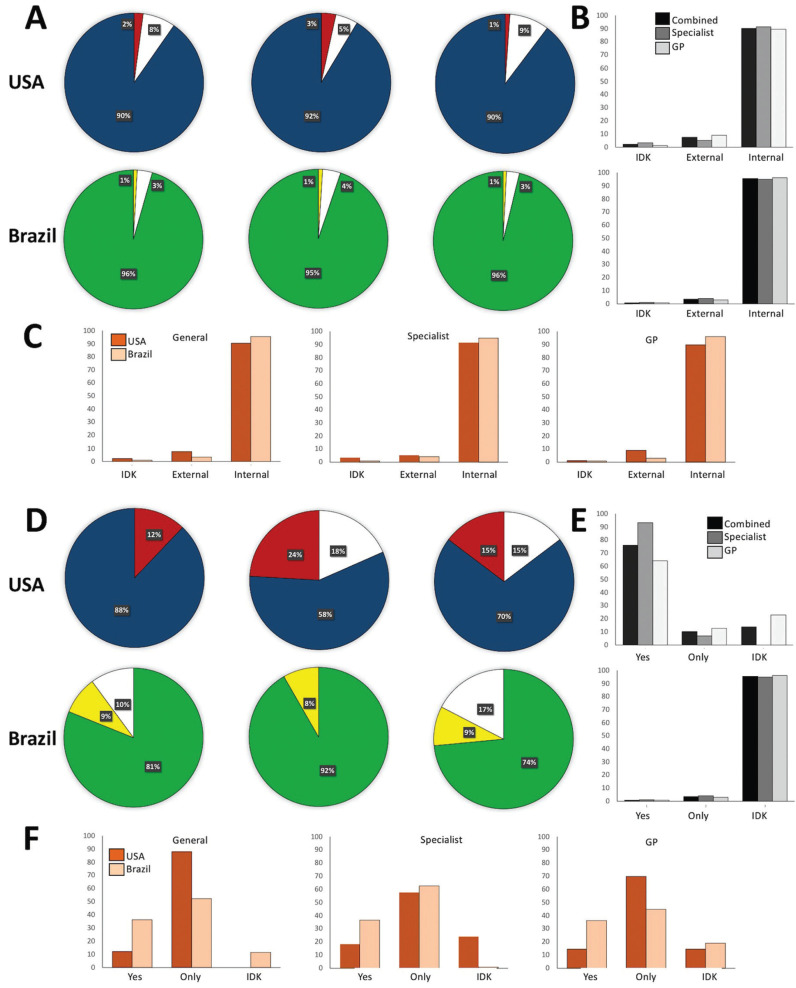
Analysis of the questionnaire about internal inflammatory root resorption through radiograph analysis. Panel A: Comparison of knowledge level between all groups (USA: top pie chart, and Brazil: bottom pie chart) on the diagnosis of external inflammatory root resorption. Blue and green are the correct answers. Panel B: The results organized by individual answer Panel C: The results comparing USA vs. Brazil. IDK (I don’t know), external, and internal root resorption. Panel D: Comparison of knowledge level between all groups (USA: top pie chart, and Brazil: bottom pie chart) on the treatment of external inflammatory root resorption. Top pie chart: USA, and bottom pie chart: Brazil. Blue and green are the correct answers. Panel E: The results organized by individual answer. Panel F: The results comparing USA vs. Brazil. Yes, only, IDK (I don’t know).

Case 2, internal root resorption (Fig. 1, Q.9): Overall 93.3% of the surveyed population gave a proper diagnosis, that comprises 93.4% of ENDs and 89.7% of general dentists. Endodontic treatment was indicated by 93.1% of ENDs and 64.4% of general dentists in cases of internal inflammatory root resorption and the use of calcium hydroxide as intracanal medicament was viewed as required (Fig. 3).

**Figure 4:**
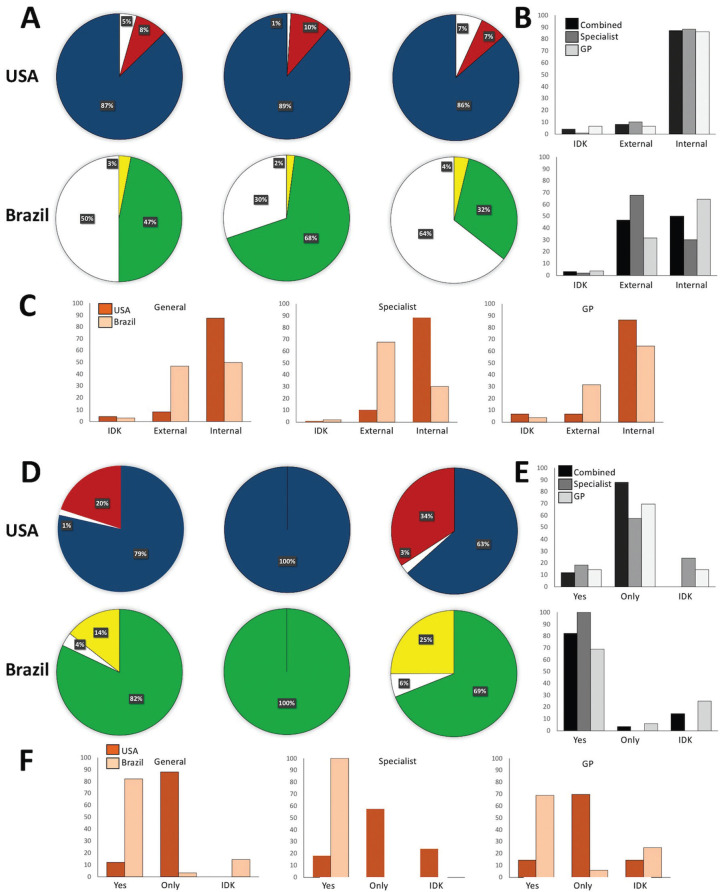
Analysis of the questionnaire about internal inflammatory root resorption through CBCT analysis. Panel A: Comparison of knowledge level between all groups (USA: top pie chart, and Brazil: bottom pie chart) on the diagnosis of external inflammatory root resorption. Blue and green are the correct answers. Panel B: The results organized by individual answer Panel C: The results comparing USA vs. Brazil. IDK (I don’t know), external, and internal root resorption. Panel D to F: Analysis of the data regarding additional exams. Panel D: Comparison between all groups (USA: top pie chart, and Brazil: bottom pie chart). Top pie chart: USA, and bottom pie chart: Brazil. Blue and green are the correct answers. Panel E: The results organized by individual answer. Panel F: The results comparing USA vs. Brazil. Yes, only, IDK (I don’t know).

Case 3 internal root resorption (Fig. 1, Q.14): The CBCT evaluation of internal inflammatory root resorption yield a correct response from 89.7% of ENDs and 83.9 % of general dentists (Fig. 4). The data are presented in Table 2.

When asked if any additional exam should be requested in cases of internal and external root resorptions, 5% of all surveyed population (6% vs. 5%) answered no, 28% (25% vs. 32%) did not know and would refer to another professional, and 144 (65%, 69% vs 60%) would request additional exams. Regarding the ENDs cohort, all 154 ENDs from both countries would request additional exams. The most common exam would be CBCT, however some professionals would request angled PAs and pulp vitality tests. All results presented in parenthesis are Brazil vs. USA within the respective cohort.

In this study, it was considered adequate when all three questions about the diagnosis and all two questions relating to the treatment were answered accurately. Statistically significant difference between the general dentists and ENDs was observed for both diagnosis and treatment of inflammatory root resorption (*p* <0.01, Table 4). Same result was observed in Brazil and USA. Years of clinical experience do not have impact on the outcome, no differences were found for both, general dentist and ENDs (*p*>0.05).

**Table 4:**
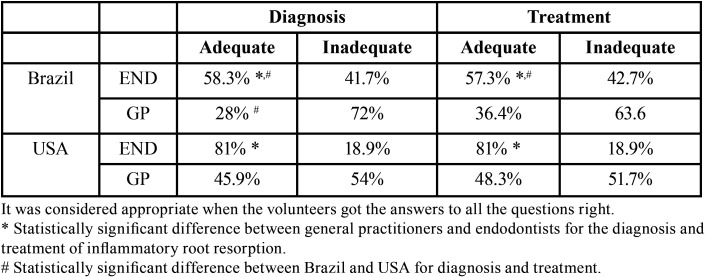
Comparison of the level of knowledge between general practitioners and endodontic specialists for the diagnosis and treatment of inflammatory root resorption.

When the responses between Brazil and the USA were compared, it was observed that both general dentists and ENDs from USA had higher adequate answers related to diagnosis (*P*<0.05). The same was observed for ENDs from USA related to treatment (*p*<0.05); however, when general dentists were compared, no differences were found between countries (Table 4).

## Discussion

This is the first article to address the difference between Brazilian and American dentists and ENDs on the diagnosis and treatment decision of inflammatory root resorptions. Our findings indicate that ENDs presented higher level of knowledge than GPs in both countries. In terms of region, USA presented higher level of knowledge than Brazil.

The better outcome observed in ENDs were expected, since they have more years of training and experience than GPs. We also observed that GPs diagnosed and chose the treatment more appropriately for internal inflammatory root resorption compared with external resorption. This can be explained by the case chosen for this study, i.e., a severe internal root resorption case, making it easier to give a proper diagnosis.

Due to a similar clinical and radiographical presentations of internal and external inflammatory resorptions, a provider should request multiple radiographs with different incidence angles (13). If a diagnosis is not conclusive, and to know its extent and severity, a 3D imaging, such as CBCT, should be used for the complete assessment of the case (14). Mastering both radiographic and clinical presentations are imperative to timely management of the lesion, limiting disease progression and, consequently, tooth structure loss.

Interestingly, the majority of GPs presented the appropriate diagnosis of internal inflammatory root resorption using PAs, however, when the CBCT was presented, higher failure of diagnosis was observed. This raises the question whether GPs know when the CBCT should be requested and, consequently, how to interpret it. Although the aim of the present research was not to compare the exams - CBCT and periapical radiograph, this surprising result was observed. Patel *et al*. in 2009 observed that although a PA radiograph is an acceptable diagnostic method for internal and external root resorption, CBCT provides a more precise information, thus, increasing the probability of a proper lesion management (11). Similarly, Madani *et al*. in 2016 demonstrated the effectiveness of CBCT imaging in root resorptions diagnosis (13). Also, Vaz de Souza *et al*. in 2016 compared the diagnostic efficacy of CBCT with parallax periapical radiographs for the detection and classification of simulated external cervical resorption lesions (15). The percentage of correct diagnoses and correct classification was around 88% and 70% for CBCT and 48.5% and 39.7% for radiography, respectively (15). It is important to point out that none of these studies examined GPs, only ENDs and radiologists and the current research demonstrated the lack of knowledge of GPs in interpreted CBCT in a case of internal root resorption.

The literature shows the need of complementary exams - such as CBCT - to correctly diagnosis. Currently, the European Society of Endodontology statement highlights the relevance of CBCT for the management of potentially restorable external resorption lesions, especially when the diagnosis is unclear, and/or treatment is being planned, beyond the follow-up. This statement is also supported by American Association of Endodontists and American Academy of Oral & Maxillofacial Radiology (16,17). However, it is extremely fundamental that the dentist know how to analyse those exams.

When the participants were questioned about the frequency of root resorption cases seen in their careers, both GPs and ENDs reported low numbers of cases overall. Epidemiological data on internal inflammatory root resorption are scarce. This can be due to its low prevalence in general population (7). However, some case studies showed that teeth with pulpitis or pulp necrosis have significantly higher prevalence of root resorption (50% and 77% respectively) when compared to healthy pulp (18). Vier *et al*. in an *ex-vivo* study observed that 74% of teeth with periapical lesions presented some degree of internal inflammatory resorption (19). External inflammatory root resorptions have shown to be of high prevalence in traumatized teeth, with a study reporting as high as 54% (20). With their elevated prevalence in pulpitis, pulp necrosis or trauma, it is worth considering that at some point, GPs and ENDs will encounter these lesions.

The difficulties in diagnosing these lesions may contribute to lower incidence reported (19), raising the need of a better training to identify these cases at early stages. For example, we observed a significant higher rate in failed diagnosis and treatment choice from Brazilian trained GPs and ENDs. A study by Hu *et al*., focused on comparing the knowledge of Brazilian GPs and ENDs on the emergency management of dento-alveolar trauma, observed an overall poor knowledge and management of the cases, and correlated that with years of graduation from dental school (21). Brazil has the highest number of Dental Schools when compared to the rest of the world (22). Most of the Brazilian’s Dental Schools are situated in areas with the highest per capita income in the country; and, despite the large number of schools, there were no improvement in the oral health of the general population in the past years (22). The lowest ratios of dentist/inhabitant, and consequently, higher number of decayed, missing, and filled teeth (DMFT) are in regions with the lowest number of schools and dentists, resulting in a lack of oral health care services in the most needed regions.

In conclusion, regardless of the country, END specialists possess a level of knowledge of the diagnosis and treatment of root resorption superior to the general practitioners. Knowledge levels of external inflammatory resorption were low for general practitioners. Comparing the results obtained in both countries, it was observed that the USA had a higher knowledge regarding these lesions that than Brazil.
